# Modelling of jet noise: a perspective from large-eddy simulations

**DOI:** 10.1098/rsta.2019.0081

**Published:** 2019-10-14

**Authors:** Guillaume A. Brès, Sanjiva K. Lele

**Affiliations:** 1 Cascade Technologies, Palo Alto, CA, USA; 2 Stanford University, Stanford, CA, USA

**Keywords:** jet noise, aeroacoustics, large-eddy simulations

## Abstract

In the last decade, many research groups have reported predictions of jet noise using high-fidelity large-eddy simulations (LES) of the turbulent jet flow and these methods are beginning to be used more broadly. A brief overview of the publications since the review by Bodony & Lele (2008, *AIAA J.* **56**, 346–380) is undertaken to assess the progress and overall contributions of LES towards a better understanding of jet noise. In particular, we stress the meshing, numerical and modelling advances which enable detailed geometric representation of nozzle shape variations intended to impact the noise radiation, and sufficiently accurate capturing of the turbulent boundary layer at the nozzle exit. Examples of how LES is currently being used to complement experiments for challenging conditions (such as highly heated pressure-mismatched jets with afterburners) and guide jet modelling efforts are highlighted. Some of the physical insights gained from these numerical studies are discussed, in particular on crackle, screech and shock-associated noise, impingement tones, acoustic analogy models, wavepackets dynamics and resonant acoustic waves within the jet core. We close with some perspectives on the remaining challenges and upcoming opportunities for future applications.

This article is part of the theme issue ‘Frontiers of aeroacoustics research: theory, computation and experiment’.

## 1. Introduction

The use of large-eddy simulations (LES) as a tool for scientific study of jet noise has expanded considerably in the last decade. It is now becoming part of the tool set being used outside of academic research, i.e. in research and development efforts directed at design and implementation of concepts aiming to reduce the emitted noise. While the cost of jet noise predictions using LES remains relatively high, the computations have leveraged the continued advancements in high-performance computing and numerical methods, and, as a result, significant strides have been made during the last 10–15 years. These have brought improved quantitative accuracy in noise predictions both in terms of the overall sound pressure level (OASPL) directivity and spectral shape for a given observer direction. The purpose of this article is to present a concise overview of the progress made using LES and draw attention to some areas where LES is now contributing to the field of jet aeroacoustics. This is not an exhaustive review—only the most salient aspects are discussed. In combination with other broader reviews of aeroacoustics and jet noise [1–3], it is hoped that the present paper provides an update on the current state of the art in LES of jets.

## 2. Progress in large-eddy simulations of jets: the last decade

### (a) Importance of the nozzle geometry and Reynolds number

The jet LES studies available in 2008 used several pragmatic compromises [4]. Most important of these was that the nozzle geometry was not directly represented in the simulation resolving jet turbulence. The effect of the jet nozzle was emulated [5,6] using Reynolds-averaged Navier–Stokes (RANS) calculation of the flow through the nozzle to generate *quasi-realistic* jet mean flow profiles near the nozzle exit. The turbulence resolving simulations started with the emulated mean flow seeded with perturbations using various approaches aiming to capture realistic jet flow turbulence. It was hoped that as the flow evolved, say after the first jet diameter or so, the physical discrepancy associated with not directly representing the nozzle and the boundary layer state at the nozzle exit accurately would be reduced, allowing comparison with laboratory measurements for the jet flow and its near- and far-field noise. As reviewed by Bodony & Lele [4], achieving a reasonable comparison with jet mean flow and turbulence measurements required rather arbitrary shifts in the axial coordinate to match the end of potential core and even after this adjustment, artefacts associated with quasi-laminar shear layers could be seen in turbulence development, and were readily evident in the radiated noise. From these simulations, it was not possible to systematically study the effect of jet Mach number, jet temperature or jet nozzle design modification, e.g. chevrons or tabs, etc.

Another important compromise was that the Reynolds number *Re* = *U*_*j*_*D*/*ν* simulated was reduced to be in the 0.1 to 5 × 10^5^ range in most of these early jet studies [4], where *U*_*j*_ is the jet velocity and *D* is the nozzle exit diameter. Without nozzle geometry, there was no high-Reynolds number wall-bounded flow to consider. The choice of reduced *Re* was made to limit the modelling contributions, based on the argument that independence on Reynolds number in the jet plume is reached for *Re*≥100 000 [7] or 400 000 [8]. While the range of spatial and temporal scales in the jet increases as *Re* increases, the large scale part remains relatively fixed, i.e. scales with *D*. At the same time, the dissipative scales shift to smaller spatial scales and broaden the Strouhal number *St* = *fD*/*U*_*j*_ range of noise to higher frequencies *f*, but may not significantly change the main jet characteristics such as peak radiation levels, OASPL directivity, etc. This does not mean that *Re* is an unimportant parameter, but rather that, above a certain value, the Reynolds number mainly affects the jet plume and radiated noise indirectly through the changes in nozzle-exit boundary layer state and early shear layer development. Furthermore, at high Reynolds numbers, the turbulence shows higher internal intermittency, i.e. the turbulent kinetic energy (TKE) dissipation rate, Kolmogorov scales etc. fluctuate more in different realizations of the flow. The upshot of this is that the probability of extreme events (which have quite a low probability), as well as high-order statistics of the turbulent flow and noise, may change with Reynolds number. This must be kept in mind when studying intermittent phenomena, such as crackle, or wave-packet intermittency.

Rather than discuss these limitations of the previous tools, we will focus on the elements which have allowed the physically realistic simulations in recent years. Nowadays, it is well recognized that the state of the nozzle-exit boundary layer is an important parameter of the jet flow development and noise radiation. Therefore, most current simulations explicitly include a nozzle at the inlet of the computational domain and are performed at realistic Reynolds number. Whether it is for laboratory jet flows or full-scale nozzles at practical operating conditions, the nozzle-diameter-based Reynolds number is typically reported over *Re* = 10^6^, which means that both the boundary layers and shear layers are likely to be transitional or turbulent, even under favourable pressure gradient. However, inclusion of the physical geometry in high *Re* flows leads to additional meshing and modelling challenges that need to be addressed without making the computational costs prohibitive. First, how to robustly generate tractable grids that appropriately capture the relevant geometric details for complex realistic nozzles? Second, how to efficiently resolve and/or model the thin turbulent boundary layer flow inside the nozzle? For laboratory jets, significant research on both topics has been undertaken and is discussed in the next two sections. As remarked by Freund [9], these efforts have culminated in simulations that represent ‘a well-constructed modelling procedure for the nozzle turbulence which can provide unprecedented sub-dB prediction accuracy with modest-scale large-eddy simulation’. Still, research is needed to extend this improved knowledge to full-scale jets from complex exhaust systems, where there are more uncertainties on how the internal engine components and favourable pressure gradient conditions affect the interior boundary layer development and potential re-laminarization [10].

### (b) Meshing and discretization

The choices of mesh topology and numerical discretization are closely linked and directly steer the dispersion and dissipation errors, especially important in aeroacoustics. Historically, four approaches have been widely used to address the mesh-generation challenge in computational fluid dynamics: multi-block structured meshes, overset grids, Cartesian (cut-cell, immersed boundary) meshes and generalized unstructured grids (e.g. hexahedral/tetrahedral/prismatic). Structured meshes with high-order spatial discretization gained early popularity in jet noise simulations [11–13], initially without the nozzle and more recently with simpler nozzle shapes [14–17]. However, treating more complex nozzle shapes, such as those with chevrons, or faceted nozzles, is challenging with this approach [18,19]. An alternative to body-fitted curvilinear meshes based on cartesian adaptive mesh refinement (AMR) provides efficient meshing of complex geometry for various approaches such as cut-cells, immersed boundary method and overset/Chimera method with embedded finer grids. The ‘LAVA’ framework developed at NASA [20,21] leverages cartesian AMR as well as its hybridization with overset curvilinear meshes. AMR on cubic volumetric elements also underpins the developments in lattice Boltzmann method (LBM) to low-Mach aerodynamics and aeroacoustics, with unmatched meshing capabilities for complex configurations. While the progress in the application of LBM to aeroacoustics is impressive [22,23], its extension to higher Mach number flows and treatment of multi-physics phenomena remain subjects of on-going research, including jet noise [24,25].

Further progress in jet computations has resulted from the use of general unstructured meshes. Shur *et al.* have pursued detached-eddy simulations (DES), LES and hybrid methods for jet aeroacoustics and broader applications in a finite volume structured grid solver ‘NTS’ with multi-block overlapping grids. They use discretizations which blend central differencing with upwinding along with different levels of turbulence scale resolving models. A different finite element based unstructured discretization underlies the ‘JENRE’ solver [26] at Naval Research Laboratory. It has been used to investigate jet noise for circular and rectangular nozzles and from military style nozzles including core plug and secondary streams [27,28], and reference therein, and by other groups [29] to evaluate propulsion airframe interaction issues for multi-stream rectangular jets with an aft-deck. At CERFACS their general purpose LES code ‘AVBP’ has been applied to jet noise simulations. Fosso Pouangué *et al*. [30] present a comparison between high-order multi-block structured and unstructured implementations, highlight the pros and cons of each and note the ability of the unstructured solver to achieve high solution accuracy at significantly lower computational cost. Recently, Abalakin *et al*. [31] have developed a general-purpose CFD solver including LES using edge-based reconstruction schemes on unstructured meshes for complex geometry. In subsequent work, Duben & Kozubskaya [32] reported promising results on jet noise including comparisons with previous results from Shur *et al*. [33].

**Figure 1.**
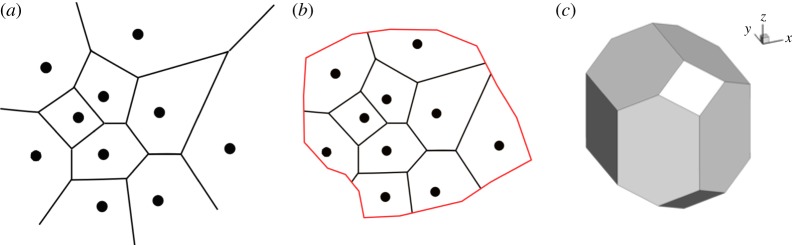
On the concept of Voronoi diagram towards three-dimensional mesh for CFD: (*a*) arbitrary set of generating points (black circles) and associated two-dimensional Voronoi diagram (black lines); (*b*) generating points and boundary surface (red lines) confining the mesh to a specific computational domain; (*c*) 14-sided polyhedra cell resulting from three-dimensional Voronoi diagram with hexagonal close packed seeding. Adapted from Brès *et al*. [35]. (Online version in colour.)

Similarly to the NTS solver, the early version of the compressible flow solver ‘Charles’ developed at Cascade Technologies used a blend of relatively non-dissipative central flux and more dissipative upwind flux on hexahedral-dominant unstructured grids with mesh adaptation. The method has successfully provided accurate jet noise predictions for a range of relatively simple geometrical configurations [34], but still lacked some of the meshing flexibility and robustness available with other approaches, like LBM, for very complex geometries. Recently, a novel mesh generation paradigm based on the computation of Voronoi diagrams has been developed within the Charles framework [35] which closed that meshing capability gap. Given a set of points where the solution is to be sampled and a description of boundary surfaces, the Voronoi diagram divides the volume based on Euclidean distance and uniquely defines a mesh. By construction, the resulting mesh possesses some desirable properties including orthogonality of face normal and cell displacement vectors. A simple example of a Voronoi diagram is provided in figure 1, along with the typical uniform 14-sided polyhedral cells resulting from the construction of the three-dimensional Voronoi diagram. Coupled with recent developments that allow for the placement of regular self-similar polyhedra (i.e. hexagonally close-packed elements) away from boundaries and the robust calculation of non-convex polyhedra in the vicinity of complex boundary surfaces, this platform allows for the generation of high-quality, body-fitted, conformal meshes suitable for low-dissipation kinetic energy and entropy preserving numerical methods [36] necessary for high-fidelity LES of multi-scale turbulent flows.

### (c) Boundary layer state at nozzle exit

The need to accurately capture the thin boundary layers at the nozzle exit in their natural state (laminar or turbulent) was deemed as a critical future step by Bodony & Lele [4]. A variety of modelling approaches have been developed in subsequent studies to capture the boundary layers at nozzle exit and thin near-nozzle shear layers. In DES and other hybrid RANS/LES approaches the nozzle interior flow is solved in RANS mode. A relatively rapid transition to a turbulent mode in the near-nozzle shear layer is desired, while minimizing acoustic artefacts. Shur *et al*. [37] have developed an enhanced DES formulation designed to promote a rapid breakdown to turbulence in shear layers. More elaborate hybrid RANS/LES approaches require further modelling in the *grey zone* where the flow transitions from RANS to LES mode.

When the nozzle interior flow is directly computed in LES, it remains impractical to fully resolve the high Reynolds number boundary layer turbulence. In many older studies, the nozzle boundary layers were assumed to be laminar, the Reynolds number was reduced and disturbances were introduced at the inlet [12] or inside the nozzle to trip the boundary layer and enable transition to turbulence. Bogey *et al.* [14,15] investigated the roles of inflow conditions and initial turbulence levels on subsonic jets at *Re* = 10^5^. The initially laminar boundary layers were tripped inside the pipe nozzle by adding low-level random disturbances uncorrelated in the azimuthal direction, with specific amplitudes chosen to achieve targeted levels of peak turbulence at the nozzle exit. As an alternative to this tuned numerical forcing, other tripping procedures inspired by roughness strips used in experiments have been suggested, including geometrical tripping [17], where a small step or serration mounted on the wall inside the nozzle is simulated, and non-geometrical tripping [38], where the first prism layers of the computational mesh at the nozzle wall are removed. While these various methods differ in costs and constraints, they all showed improvements of the flow and noise predictions towards experimental measurements.

**Figure 2.**
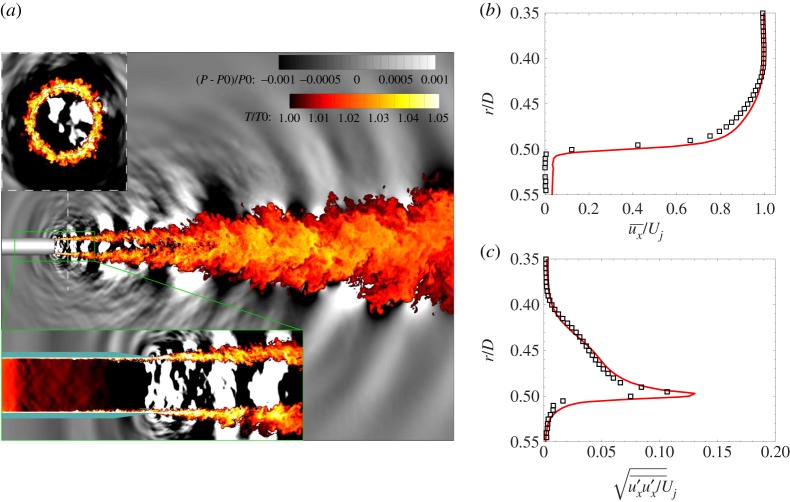
LES of an isothermal Mach 0.9 turbulent jet featuring fully turbulent nozzle-exit boundary layers: (*a*) visualization of the instantaneous pressure and temperature fields; nozzle-exit profiles of the time-average (*b*) and rms (*c*) of streamwise velocity from experiment (black square) and simulation (solid line). Adapted from Brès *et al*. [39]. (Online version in colour.)

**Figure 3.**
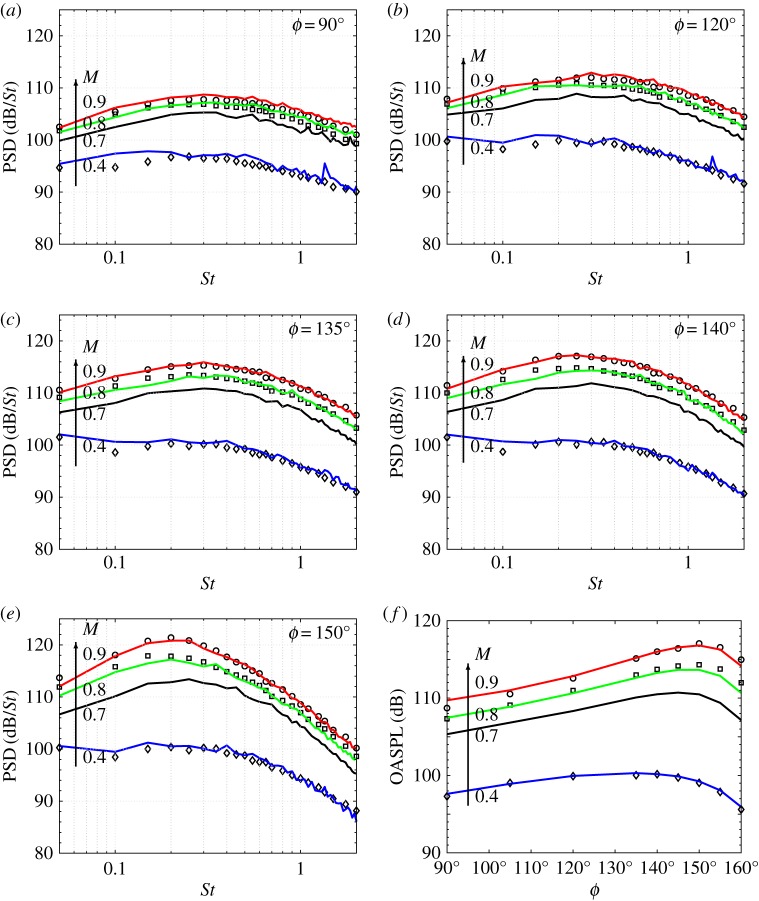
(*a*–*f* ) Comparison of the noise spectra and directivity on a cylindrical microphone array of radius 14.3*D* for subsonic turbulent jets at different Mach numbers *M* = 0.4 to 0.9: experiment (symbols); LES (lines) with near-wall mesh refinement, synthetic turbulence and wall modelling on a 16M mesh. (Online version in colour.)

While the early simulations with the Charles solver were also performed at reduced *Re* with initially laminar boundary layers [34], later efforts have focused on how to further improve the prediction of the turbulent flow inside the nozzle at full Reynolds number in a flexible, robust and cost-effective way [39]. The modelling approach pursued is a combination of synthetic turbulence seeding to enable transition, localized near-wall mesh refinement to provide sufficient grid resolution within the boundary layer to resolve the outer scale turbulent eddies and wall-stress modelling method [40–42] to model the unresolved scales near the wall. With the exception of the early attempt by Andersson *et al*. [43], wall-modelled jet LES has only recently received renewed interest [44] and has now become a topic of significant research [45–47]. As shown in figures 2 and 3, and discussed in more detail in Brès *et al*. [39], the combined approaches were initially applied to an isothermal Mach *M* = *U*_*j*_/*c*_∞_ = 0.9 turbulent jet at *Re* = 10^6^ and led to fully turbulent nozzle-exit boundary layers with significant improvement of the flow field and noise predictions. Subsequently, the study was extended to a range of Mach *M* = 0.4, 0.7 and 0.8 for the same converging-straight pipe nozzle, with similar accuracy in flow and noise predictions. All the simulations were performed in close collaboration with a companion experiment at Pprime Institute in Poitiers (France), and matched the experimental Reynolds number. Combined with appropriate resolution in the shear layers and jet plume (see next section), these modelling approaches resulted in sub-dB prediction accuracy for most relevant inlet angles *ϕ* and frequencies *St* = *fD*/*U*_*j*_ (up to *St* ≈ 2 for grids of modest size, as low as 16 million cells).

#### (i) Turbulence in jet shear layers and plume.

As already discussed, the turbulent jet flow which emerges from a nozzle begins with relatively thin shear layers. If the nozzle interior boundary layer is turbulent the near-lip shear layer is naturally turbulent. For laminar exit flow the shear layer becomes turbulent rapidly due to instabilities whose wavelength scales with the initial shear layer (momentum) thickness. If the flow at nozzle exit is already turbulent, the TKE and streamwise rms velocity *u*′ increase monotonically with distance as the shear layer spreads. For laminar shear layer, however, an overshoot in *u*′ along the lipline occurs due to transition and then approaches values consistent with turbulent shear layer self-similarity. Brès *et al*. [39] report these distinct trends in simulation results as the nozzle interior flow modelling is altered to produce laminar versus turbulent jets. Interestingly, they also found that the initially laminar jet results in an increase in the far-field radiated sound by as much as 3–4 dB at high frequencies (those corresponding to the shear layer dynamics), compared to the initially turbulent jet. This effect was previously reported by Bogey & Bailly [14,15] in their studies of jet noise for flow emerging from a straight pipe with different degrees of disturbances within the boundary layer. Linear stability analysis of the near-nozzle mean-velocity profiles suggests that the differences in radiated noise between the initially laminar and turbulent jets are related to the differences in growth rate of the Kelvin–Helmholtz (KH) mode in the near-nozzle region [39]. Therefore, it is important to ensure proper resolution of the shear layers as they grow, specifically along all three spatial directions since the turbulent eddies in a shear layer are not much elongated unlike the near-wall turbulent eddies. In practice, this means maintaining a relatively isotropic grid in the turbulent shear layers and the jet plume downstream of the potential core, which is typically easier to achieve in the unstructured mesh setting.

#### (ii) Far-field noise predictions.

Prediction of far-field radiated noise from jet flow LES requires a hybrid approach, where the important scales of turbulence within the noise-producing region of the jet are resolved, and the propagation of the small amplitude acoustic fluctuations from the near-field source region to the far field is computed analytically. The Ffowcs Williams–Hawkings (FW–H) equation [48] is one of the most commonly used hybrid methods. More complex models for wave propagation, such as linearized Euler equations (LEE) [15] or their full nonlinear version [49], can also be integrated numerically for near-field acoustics. When the acoustic field is intense, as in sonic boom propagation [21], or in crackle emission from hot supersonic jets, discussed in §§3b, the nonlinear effects are important.

In the FW–H approach, the turbulent flow volume is surrounded by an acoustic data surface, i.e. FW–H surface, on which the time-varying data is saved as the jet LES is computed, and the quadrupole contributions from the volume-distributed noise sources outside of the FW–H surface are neglected. In a post-processing step, the far-field radiated sound is calculated using the FW–H surface data via analytically known Green's function for a stationary or uniformly moving ambient medium. The placement of FW–H surface, and the spatial and temporal resolution of the data saved on FH-W surface are important. If the FW–H surface lies inside the turbulent flow, the data collected on it do not include the sound produced by the turbulent flow lying outside of it and (vigorous) crossing of turbulence across the surface is a contributor to spurious acoustics. On the other hand, if the FW–H surface is placed too far away from turbulent jet, predictions based on it might also be incorrect because the *acoustic data* recorded on that distant surface might be corrupted by significant numerical errors. In particular, Bodony & Lele [4] pointed out that there was some debate on how to treat the downstream outflow surface in the wake of the jet, namely, whether to close it or leave it open to avoid the spurious sound generated by vortices passing through that surface, which is a by-product of the quadrupole contributions being neglected. Shur *et al*. [5] introduced a different approach, referred to as the method of ‘end-caps’, in which the complex far-field pressure predicted from several FW–H surfaces with the same shape but outflow disks at different streamwise locations are phase-averaged. This leads to the reduction or cancellation of the uncorrelated noise produced by the turbulent eddies crossing the successive outflow disks. This robust and reliable method has been shown to yield better far-field noise predictions with minimal performance penalty [34,50] and is now being widely used. More recently, Ikeda *et al*. [51] proposed an alternative approach where a quadrupole contribution correction under frozen turbulence assumptions is added to the surface integral on a single end cap. They argued that the approach provides similar results to the method used by Shur *et al*. [5], with no tunable parameters, and only small additional cost compared to the conventional permeable-surface integrals.

## 3. Large-eddy simulations as a complement to experiments

One way that LES are contributing to a better understanding of jet noise is as a complement to experiments, in particular, for tactical exhaust systems. Detailed measurements in full-scale engines are costly and difficult, and most laboratory facilities are limited to smaller-scale jets at lower temperatures. For such high-speed heated jet from realistic nozzle configurations, LES can arguably provide insight on the jet flow field and acoustic field in a more flexible and cost-effective way than most *in situ* or laboratory testing.

### (a) Leveraging temperature effects towards noise reductions

To investigate the impact of inlet temperature non-uniformity on the jet flow and noise, LES were performed with the Charles solver for heated over-expanded supersonic jets issued from a faceted military-style nozzle [52]. The numerical study was motivated by recent experimental efforts at Virginia Tech University [53] where a noise mitigation concept based on non-uniform temperature profile is being investigated. In these experiments, temperature non-uniformities are produced through auxiliary nozzle hardware that introduces a stream of colder fluid inside the exhaust system, upstream of the nozzle exit. While the laboratory configuration is limited to low temperatures and low-temperature ratios, three different high-temperature engine operating conditions were considered in the LES. The first condition simulated is a flow at military power settings with nozzle temperature ratio NTR = 3, i.e. the ratio of total to ambient temperature. The second case is a flow at an afterburning condition with NTR = 7, to characterize the noise increase with regard to temperature. Finally, the third condition corresponds to a flow with non-uniform temperature profile, consisting of an annulus of afterburned exhaust and a central stream of military power exhaust, which is compared to the afterburning condition to assess potential noise reduction. This scenario could potentially be achieved in high-performance exhaust systems if, for instance, only the outer rings of the afterburner are activated.

**Figure 4.**
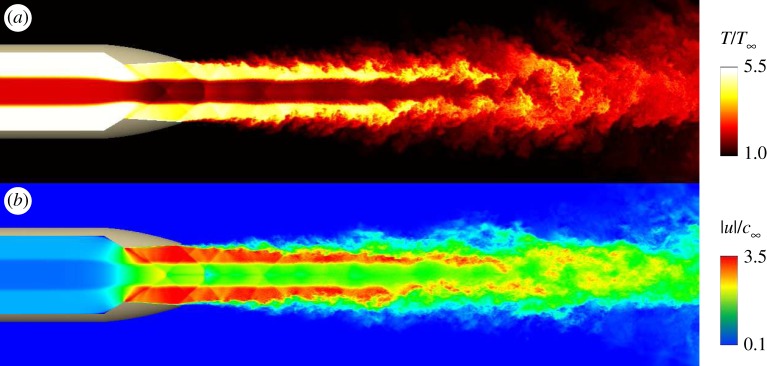
Instantaneous temperature (*a*) and velocity magnitude (*b*) for an over-expanded supersonic jet issued from a military-style nozzle with non-uniform inlet temperature (i.e. annulus of highly heated afterburned exhaust and central stream of hot military power exhaust). Adapted from Brès *et al*. [52]. (Online version in colour.)

**Figure 5.**
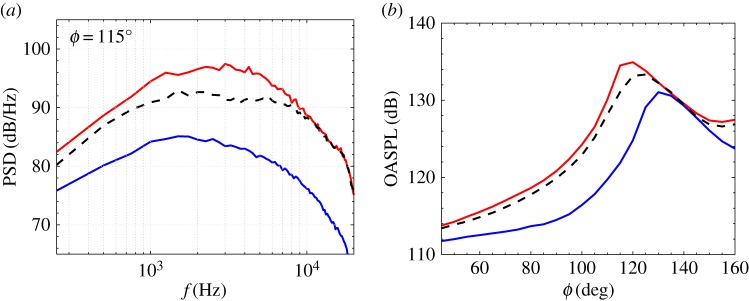
(*a*) Noise spectra and (*b*) OASPL directivity for the hot over-expanded supersonic jet from a military-style nozzle at different operating conditions: military power (solid blue line); afterburner (solid red line); non-uniform inlet temperature (dashed line). Adapted from Brès *et al*. [52]. (Online version in colour.)

Figure 4 shows a visualization of the instantaneous temperature and velocity for the non-uniform temperature conditions. Leveraging the improved meshing and wall modelling capabilities discussed in §2, the sharp-throat converging-diverging faceted nozzle is explicitly included in the computational domain and the flow field features thin turbulent nozzle-exit boundary layers and initially turbulent shear layers. For these over-expanded conditions, a complex but relatively steady shock train is present, starting at the sharp nozzle throat and extending in the jet plume several nozzle diameters downstream of the nozzle exit. All the simulations have the same nozzle pressure ratio and there are no changes in nozzle geometry, even for the afterburner conditions. These assumptions lead to the same thrust for all cases and result in similar shock structure in the jet. By contrast, there are significant changes in radiated noise, as shown in figure 5. As expected, for the two highly heated cases with afterburner flow, the increase in jet velocity drives the increase in noise over a wide range of frequencies and angles. However, for the non-uniform condition, the radiated noise is noticeably reduced compared to the afterburner conditions, in terms of peak OASPL, radiated power and spectra over a large frequency range and for most inlet angles *ϕ*. The results of this proof-of-concept LES study indicate that there is merit to the idea pioneered in experiments at lower temperature when taking into consideration more realistic conditions. Much work remains to fully analyse the LES data generated by this investigation, along with more concepts worthy of examination to improve the simulations and noise mitigation.

### (b) Crackle

**Figure 6.**
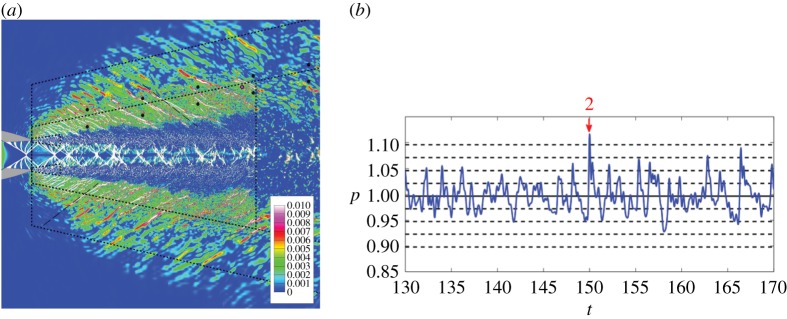
Simulations of hot supersonic jet emitting crackle: (*a*) contours of a flow sensor depicting instantaneous shock waves, including shocklet emission in the region in between large scale eddies. Thick dashed lines mark the region of mesh refinement and the oblique line with long dashes shows the Oertel Mach angle expected for this operating condition. Adapted from Nichols *et al*. [59]; (*b*) an example of near-field pressure-time trace depicting crackle. The pressure is normalized by the ambient pressure and each dashed line indicates deviations corresponding to one standard deviation from the mean value. Adapted from Nichols *et al*. [58]. (Online version in colour.)

Ffowcs Williams *et al*. [54] identified crackle as the most annoying component of the noise radiated by supersonic hot jets. They associated it as an intense intermittent sound with a pressure wave form consisting of a rapid pressure rise and gradual return, akin to a sonic boom, radiated in the direction of the most intense jet noise. They showed that pressure signal skewness greater than 0.4 was associated with crackle. In the laboratory experiments, it was not possible to identify the mechanism responsible for crackle emission, but based on the pressure amplitude they estimated that nonlinear steepening, i.e. acoustic shock formation via progressive steepening of an initially smooth waveform, would require quite a long distance. In subsequent years, crackle has been detected in laboratory measurements and field tests of military engines [55]; its source mechanism and the role of nonlinear steepening were not fully understood. While steep shock-like near-field acoustic waves had been observed in the relatively low Reynolds number simulations of hot supersonic jets [56,57], the comprehensive LES of hot supersonic jets by Nichols *et al*. [58,59] is a good example of LES complementing laboratory experiments. The simulation included sufficiently fine grid to resolve steep waves in the near-field. The LES results were first validated against far-field noise from laboratory tests at NTR = 3.65 (i.e. the largest nozzle temperature ratio tested experimentally), which allowed some confidence in the detailed near-field exploration of the noise emission process, and then extended to higher temperatures, up to NTR = 4.5. Through these studies, Nichols *et al*. have shown that crackle is emitted as a weak shocklet, whose shock-like signature can be traced all the way to the eddying motions in the jet shear layer. They also noted that high-frequency shocklets are emitted from the near-nozzle shear layer, consistent with Mach wave emission from eddies moving at Ortel convective Mach number *M*_co_ = (1 + *M*_*j*_)/(1 + *c*_∞_/*c*_*j*_); however, the intense crackle emissions were found to move more rapidly and were relatively infrequent as shown in figure 6. Recent simulations of hot supersonic jets [27,60] have also indicated the presence of crackle but not focused on its emission. Tam *et al*. [61] stress that source nonlinearity does not mean that nonlinear propagation does not exacerbate the problem. Such effects complicate the inversion of near-field pressure signals back to their source and seem evident in recent analysis [62] of F-35 noise measurements. Further exploration of these problems combining simulations, test data and nonlinear propagation models is warranted.

### (c) Shock associated noise, screech and impingment tones

Harper-Bourne & Fisher [63] interpreted the broadband shock-associated noise (BBSN) in pressure-mismatched jets as an outcome of the interference between localized point-sources at the locations of the interaction between the shear layers and the shock system in the jet. Tam [64,65] formulated this interaction problem and proposed a solution of the pressure field in terms of parameters expressing the strength of the shock-cell modes and shear layer instability waves. Using this, Tam also proposed semi-empirical formulas for predicting BBSN spectra and its directivity.

In recent years LES of hot supersonic jets including over-expanded and under-expanded conditions have been investigated by many groups. These studies have included axisymmetric, chevroned and rectangular nozzles and cold, heated and highly heated conditions. While most effort has been devoted to the validation of the LES predictions [34], some analysis of the BBSN source mechanisms is also reported. Shur *et al*. [33] studied BBSN for single and dual-stream sonic under-expanded jets and showed the effectiveness of their simulation methodology in capturing the specific signatures of BBSN including the effect of the flight stream. Their study revealed the mechanism behind the new BBSN features in dual-stream flows with under-expanded secondary stream and subsonic core stream [66], although the associated spectra were overpredicted. Later Suzuki [67] analysed the results more deeply and suggested that the model of cross-spectral density introduced by Harper-Bourne & Fisher can be reconciled with instability wave packets of different azimuthal index, but the far-field noise spectra predicted by such a model do not closely resemble the measurements, or the FW–H surface prediction from full LES. On the other hand, a statistical source model based on RANS by Morris & Miller [68] better captures the BBSN spectral shapes. Evidently, possibilities for further improving the modelling of shock associated noise remain open. Liu *et al*. [69,70] study pressure-mismatched round jets matching experiments at the University of Cinncinati, and probe the pressure disturbance behaviour in wavenumber-frequency domain, cross-correlation analysis in the shock-cell region, and convection velocity of disturbances in the flow. Specific BBSN model improvement was not conducted. Later work has considered more realistic nozzle geometry and heated flows. In a recent study, Arroyo *et al*. [71] applied wavelet analysis to LES data to isolate the effect of different azimuthal modes on BBSN noise radiation. Evidently, much ongoing research is devoted to the noise of pressure-mismatched jets, and one can anticipate new insights and models to emerge from these simulation efforts.

Tam [72,73], and Raman [74] have provided a critical review of jet screech research (also see [75] for an update). They note that existing models of screech are capable of predicting the screech tone frequency, following the notion of a screech feedback loop pioneered by Powell [76], but no simple models capable of predicting the screech amplitude exist as yet. This observation motivated the study of a simpler model problem [77–80]. An upshot from these studies is the discovery of the shock-leakage phenomena relevant for screech noise emission at high amplitudes [78,80]. Under appropriate conditions, i.e. when the instantaneous local vorticity is sufficiently weak, the shock wave system which is normally trapped within the supersonic jet core leaks out and emits a wave into the ambient medium outside the jet. While recent LES of pressure-mismatched jets [81] have shown that the screech phenomena can be captured in high-fidelity simulations, there are, at present, no comprehensive studies of screech dynamics using LES which reproduce and explain the nonlinear phenomena observed in laboratory experiments, such as the variation of screech intensity, mode staging and role of screech in twin jet coupling.

Recent research on screech and other resonant acoustic phenomena (see §§4b), such as impingement tones, has indicated an alternative mechanism for closing the feedback loop besides the upstream travelling acoustic waves envisioned by Powell. Following the discovery of an upstream propagating hydrodynamic mode by Tam & Hu [82] in supersonic jets the role played by such modes in impingement tones has been highlighted by Bogey & Gojon [83,84] through analysis of LES data, and by Edgington-Mitchell *et al*. [85] in jet screech via sophisticated analysis of experimental data. Many aspects of the problem, however, remain open, including nozzle receptivity and possible co-existence of multiple feedback and receptivity paths. Recent experiments on impinging supersonic jets by Weightman *et al*. [86,87] detail multiple-source mechanisms and multiple-receptivity paths whose manipulation leads to changes in the dominant resonant mode of the jet. Capturing and explaining the rich dynamical behaviour of such coupled flow acoustic problems remains a topic for future research.

## 4. Large-eddy simulations as an aid to jet modelling

Since Lighthill [88] introduced the acoustic analogy in the 1950s, some significant advances have been made in modelling sources of noise in turbulent jets. This is true in particular for academic, circular, subsonic jets, while complex nozzles and pressure-mismatched conditions typically require modifications to the semi-empirical approaches. Arguably, the most important recent progress has been made in the modelling of wavepacket dynamics [1], the understanding of trapped resonant acoustic waves [89] and the development of a generalized acoustic analogy [90]. As discussed in the next three sections, LES is starting to play a key contributing role in these modelling efforts, as a provider of high-resolution, three-dimensional, time-resolved flow data to guide and test the models.

### (a) Wavepacket dynamics

As reviewed by Jordan & Colonius [1], there have been great advances in the past few years in identifying the physical mechanisms underlying the peak jet noise radiation to aft angles—namely the existence of convective, large-scale wavepacket structures that directly radiate to the far-field. While the observation of wavepackets and the corresponding theory date to the earliest days of jet noise research [91,92], the recent breakthroughs were made possible in large part by progress in experimental microphone arrays diagnostics, in high-performance computations with LES, and in spectral analysis tools.

One spectral approach ideally suited for turbulent jets is the frequency domain version of spectral proper orthogonal decomposition [93], referred to as SPOD [94]. As described by Schmidt *et al*. [95], SPOD ‘identifies energy-ranked modes that each oscillate at a single frequency, are orthogonal to other modes at the same frequency, and, as a set, optimally represent the space–time flow statistics’. SPOD combines advantages of the usual spatial version of POD and dynamic mode decomposition [96], and identifies flow structures that evolve coherently in space and time.

Another frequency-domain technique called *resolvent analysis* has emerged from dynamical system theory. This analysis of turbulent mean flows is based on the assumption that large-scale coherent structures can be modelled as responses of a linear operator to stochastic forcing [97]. Most recently, the method has proven to yield accurate low-order representations of the jet acoustic radiation [98] and demonstrated its capability to identify resonance mechanisms in turbulent jet flows [99].

**Figure 7.**
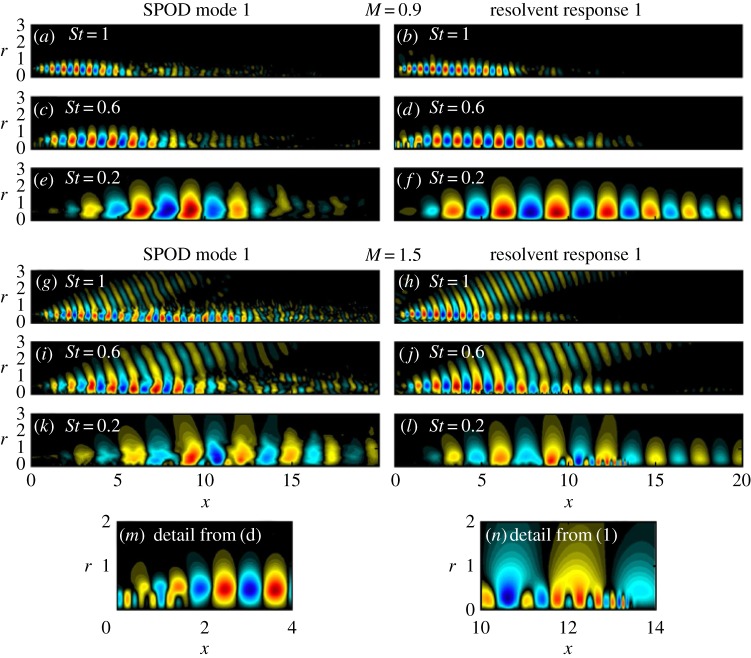
Resolvent response modes (right) compared to SPOD modes (left) educed from LES data computed with Charles. The fluctuation pressure is shown for a transonic Mach 0.9 jet (*a*–*f* ) and an ideally expanded supersonic Mach 1.5 jet (*g*–*l*). The nozzle is located in the *x* = 0 plane and the spatial coordinates are normalized by the jet diameter *D*. The component with azimutal wavenumber *m* = 1 is shown for three representative frequencies expressed in terms of the Strouhal number *St* = *fD*/*U*_*j*_. Panels (*m*,*n*) zoom in on the different resonance mechanisms present in the two jets (see §§4b). Adapted from Schmidt *et al*. [95]. (Online version in colour.)

As part of an ongoing collaborative effort to improve understanding and modelling of the turbulent sources of sound in high-speed jets, extensive LES databases have been generated with the Charles solver for a range of jet conditions and nozzle geometries [39,95], including the Mach 0.9 turbulent jet presented in figures 2 and 3. The dominant SPOD modes were educed from the unsteady LES data, and resolvent analysis computed optimal responses to stochastically forced linear systems, based on the time-averaged LES flow field. For each response mode, the method also computes a corresponding optimal forcing distribution. In the context of turbulent flows, these resolvent forcing modes can be associated with nonlinear interactions as well as stochastic inputs to the flow, for example the turbulent boundary layer in the nozzle that feeds the jet. The resolvent response modes, on the other hand, predict the most energetic large-scale coherent structures in the turbulent flow. In jets, these coherent structures take the form of convective wavepackets that directly radiate sound to the far-field. Figure 7 shows the wavepackets that were educed from the LES of a subsonic turbulent jet and an ideally expanded supersonic turbulent jet using SPOD (left), and their predictions by the resolvent analysis (right) for the same frequencies. In general, good agreement is found between the simulation data and resolvent predictions. Of particular interest for noise modelling are the Mach wave radiation patterns in the *M* = 1.5 case seen in figure 7*g*–*j*, which are at the root of aft-angle supersonic jet noise, and the waves highlighted in figure 7*m*,*n*, which identify different acoustic resonance phenomena (see §§4b).

Overall, the studies of the LES data by Brès *et al*. [39] and Schmidt *et al*. [95] highlight some key features of turbulent jets. First, decomposition of the radiated noise into azimuthal Fourier modes confirmed that the axisymmetric mode is the dominant source of sound at the peak radiation angles and frequencies, and that the first three azimuthal modes recover most of the total acoustic energy. Second, over a range of frequencies and these first few azimuthal modes, turbulent jets in both subsonic and supersonic regimes exhibit a *low-rank* behaviour, that is linked to KH-like wavepackets in the initial shear-layer. Here, low-rank means that the physical mechanism associated with the first, most energetic mode is prevalent. At other frequencies, the response is not low rank and consists of a combination of similarly amplified modes, with both KH-type and Orr-type wavepackets, which are primarily active downstream of the potential core. These findings have important implications for improving wavepacket modelling and help explain why some of the early models [100–102] failed at very low frequencies.

### (b) Trapped acoustic waves and resonance

Besides its contributions towards the understanding of the most energetic large scale coherent wavepacket structures that dominate the shear layer, the *M* = 0.9 LES database [39] was also fundamental in uncovering and modelling a novel class of resonant acoustic waves that are trapped within the potential core of the jet. As discussed in detail by Towne *et al*. [89] and Schmidt *et al*. [99], these waves experience the shear-layer as a pressure-release duct and are therefore radially confined to the very near-field of the jet. At certain frequencies, the trapped waves resonate due to repeated reflection between end conditions provided by the nozzle-exit plane and the streamwise contraction of the potential core. With guidance from the LES data, Towne *et al*. [89] developed a cylindrical vortex sheet model and analytic dispersion relations that predict the existence of these waves and resulting resonance for isothermal jets within a Mach number range of 0.82 < *M* < 1.0. Indeed, at *M* = 0.9, both the upstream and downstream-propagating waves were observed in the jet core in the simulations, as well as the corresponding discrete tones in the near-field acoustic pressure very close to the nozzle exit for the LES and the companion experiments. While it had been postulated [82] that compressible jets can support hierarchical families of modes representing upstream and downstream travelling acoustic waves (in addition to the classical KH modes), these trapped waves decay rapidly away from the jet and had not been systematically observed and characterized until now. A number of subsequent studies have demonstrated the relevance of the trapped acoustic waves in several other resonance phenomena such as jet-flap tones [103] (i.e. when the flap on a wing is positioned close to a subsonic jet), screech tones [85,104] and impinging tones [83,105]. Additional simulations and modelling efforts are ongoing to gain a more a complete understanding of the dependence of these waves on relevant jet parameters such as Mach number, temperature ratio, nozzle geometry, flight effects, etc.

### (c) Acoustic analogy

LES-informed modelling of aeroacoustic sources, and in particular for jet noise sources, has been anticipated at least since the 1980s [106]. The development of reliable LES databases covering a range of jet operating conditions and nozzle geometries had to wait until the present decade. While further validation of LES databases, in terms of turbulence associated source-covariances, is required in future, several parallel efforts in modelling jet noise sources are ongoing and are reviewed thoroughly in other contributions contained in this special volume. We will restrict ourselves to a brief summary here. Karabasov *et al*. [107] and the follow-on studies (see [108,109] and references therein) adopted Goldstein's generalized acoustic analogy (GAA) [90], i.e. the Reynolds stress fluctuation covariances, enthalpy flux fluctuation covariances and the cross-covariances with the former, for modelling jet noise sources. This recognizes the role played by the non-uniform jet mean flow in the linear operator and allows a consistent treatment of flow-effects on sound generation and propagation. Extensions to incorporate more efficient evaluation of far-field noise, asymptotic reformulation for slowly spreading shear flows at low frequency, co-annular streams, non-isothermal flows and shock-associated noise, etc. have been undertaken. GAA source covariances have also been analysed by Bassetti & Nichols [110] and others [111,112] using jet LES data. As the newly developed methods are evaluated over a wide range of jet flow conditions and geometries, a more clear sense of the benefits of the improved models (including their simplifications) would emerge. It suffices to underscore that significant improvements in the prediction of noise directivity and changes in the noise spectral shape (as a function of polar angle) are already evident from the results.

## 5. Remaining challenges and upcoming opportunities

Looking back at the list of key open issues identified by Bodony & Lele [4] in their review of jet LES a decade ago, a few items have been mostly addressed, i.e. inclusion of nozzle geometry, closing of the FW–H surface, comparisons between computation and experiment. However, some challenges remain, in particular in terms of sub-grid scale (SGS) modelling and limited frequency bandwidth of the noise predictions.

For all mesh topologies, the interplay between the modelling choices regarding the treatment of subfilter scale motions and the numerical bandwidth of the discretization schemes used, determines the effective bandwidth of the simulation results. While comprehensive cross-comparisons across different LES solvers and modelling of fine-scale motions are still lacking, some observations can still be made. Simulations with schemes employing minimum numerical dissipation require SGS modelling to prevent tail up of energy at the shortest scales [39]. In this context, the use of the SGS model was found to be crucial for accurate prediction of high *Re* wall-bounded turbulence such as nozzle-interior boundary layers. Turning off the SGS model yielded erroneous profiles for turbulent fluctuations and also incorrect boundary layer profile [113]. Numerical approaches which already have significant built-in numerical dissipation due to either upwind-biased stencils, upwinded fluxes, artificial dissipation, or explicit filtering, often show further dissipation of the shortest scales when additional SGS model is included [114,115]. Furthermore, in high Reynolds number flow applications, it is impractical to resolve the viscous scales near walls, requiring wall models and near-wall grids scaled by the local (outer) boundary layer scale. The solution is also impacted by the boundary condition imposed on the wall-modelled large-eddy simulation (WMLES) solution. These are topics of active research [40,116], and their impact on aeroacoustic predictions are yet to be explored in detail, as is the impact of the additional modelling associated with hybrid RANS-LES approaches. A comprehensive assessment of the pros and cons of various modelling and numerical choices in treating sub-grid processes awaits future studies, and is perhaps a suitable theme for a focused workshop. It suffices here to underscore the need to carefully examine the grid resolution being used in various flow regions and its implication in terms of the (effective) dispersion and dissipation properties of the numerical algorithms. This includes boundary conditions, interface treatment, turbulent inflow generation or forcing, sponges, etc. as well as the implications of other modelling assumptions such as explicit subgrid modelling or implicit LES, hybridization with RANS (whether just at walls as in WMLES, or in DES-style).

The other concern raised by Bodony & Lele [4] was that the frequency bandwidth for the predictions was restricted to *St* = 1.5 − 3. While this frequency range is sufficient for supporting the modelling effort of the peak noise radiation, acoustic resonance and wavepacket dynamics [39,89,99], larger bandwidth is likely needed to meet regulation requirements for industrial use at full scale. Over the past decade, there has been some limited progress on this issue, mostly achieved through the inclusion of the nozzle geometry and better capturing/modelling of the small but energetic eddies in the nozzle boundary layers and early stages of the jet shear layer development. To further improve the high-frequency noise predictions, the typical approach is to increase the grid resolution in the shear-layers and near-nozzle region. For instance, Brès *et al*. [39] conducted a grid resolution study for the *M* = 0.9 turbulent jet previously discussed and showed that the frequency limit was increased from *St* ≈ 2 to 4 by doubling the resolution in the jet plume in all directions. For the refined LES case, the unstructured mesh contained 69 million cells (up from 16 million), the simulation time step was decreased by half (because of CFL constraints) and the CPU cost (normalized to the same total simulation time) was increased 10-fold. Overall, this approach comes with a significant increase in computational cost, only partly mitigated by advancements in high-performance computing, and it is still unclear if tractable resolution can yield sufficiently high bandwidth predictions for practical applications. As an alternative to (or in combination with) finer grids, Bodony & Lele [4] suggested the development of an ‘SGS noise model which aims to estimate the missing noise from the unresolved scales using information from the resolved scales’. Preliminary steps towards such models were also taken by them utilizing the *generalized acoustic analogy* approach [90] applied to subfilter scales [117,118]. The availability of LES databases where jet noise sources have been resolved over a significant frequency bandwidth should now enable further advancement of such models. Additionally, the approach of subfilter scale enrichment [119] and stochastic source modelling [120,121] could also be used to extend the bandwidth of the predicted noise.

In terms of research opportunities, prediction and reduction of the noise radiated by hot, supersonic jets has been a traditional research driver. Recent studies of strongly heated supersonic jets [60,122] underscore the effect of modelling variations in the specific heat capacity accurately. Further model developments for jet LES, including combustion modelling at an appropriate level, simulations with multi-component gas mixtures (combustion products and air), and more complete multi-physics modelling including heat-transfer and cooling flows, can be envisioned for specific applications. In a laboratory set-up, it becomes quite challenging to duplicate theses engine operating conditions, especially the full power with afterburner condition of military engines. This places limits on hardware testing of potential noise reduction technologies. Once sufficient confidence has been gained in the predictive power of LES, it can support noise reduction concept evaluation and significantly reduce the required hardware testing. Coordinated simulation (RANS and LES) and laboratory testing have been carried out during the last decade [123,124] and such efforts are expected to intensify in future.

The trend towards higher bypass ratio, larger engines for commercial aircraft has elevated the importance of installation effects. Tyacke *et al*. [125] have developed a multi-fidelity hybrid RANS-LES approach for simulations of an installed aero-engine. They use the immersed boundary approach to incorporate additional geometrical details such as the engine pylon and a body force method to introduce the effects of the fan and OGV wakes. Propulsion systems which are closely integrated with the airframe are being envisioned for new advanced civilian and military aircraft. Over the wing engines, distributed propulsion, twin jets [126], non-circular inlets with serpentine ducts [127], multi-stream rectangular nozzles, aft-deck, etc. are some of the concepts being evaluated for engine performance and noise reduction potential, with additional benefits for thrust vectoring and signature reduction. Complex engine flow paths and their alteration bring up the need for more comprehensive simulations of combined aero-thermal-structural systems. Thermal and acoustic fatigue life of components becomes another research driver. Modelling and capturing the essential details of the engine geometry and flow path is essential for reliable predictions of performance and noise of the new concepts at an early stage of development. While significant progress has been made with wall modelling of the internal flow, many research questions remain, in particular when it comes to the model's ability to predict hot streaks, heat transfer and separated/reverse flow in part of the complex exhaust system upstream of the nozzle.

Prediction of the vibro-acoustic environment associated with rocket launch has driven considerable research in recent years [21]. Such capabilities are being leveraged for other aeroacoustic problems including airframe and landing gear noise, and rotorcraft and urban air mobility (UAM) concepts. Other major opportunities where LES could impact the R&D efforts include rocket launch transient load mitigation including water sprays, launch abort system safety evaluation, and retro-rockets in the design of entry/descent/landing systems for large martian vehicles.

In terms of high-performance computing, the current trend is towards mixed architectures and incorporating some GPU computing for the jet simulation will likely be necessary to leverage these enhancements. Indeed, the Oak Ridge National Laboratory has recently announced the selection of Cray and AMD to provide the laboratory with its first exascale supercomputer for 2021 deployment. Poised to deliver greater than 1.5 exaflops of HPC and AI processing performance, the *Frontier* system will feature future-generation AMD CPUs and Radeon GPUs. Some jet simulations have already been performed on GPUs by Markesteijn, Semiletov & Karabasov (see [128] and the follow-on studies [129] with references therein) and showed promising results in terms of performance. The authors report that the GPU solver was able to handle computational grids up to 80 million grid cells on a conventional desktop computer equipped with a few GPUs, while obtaining acoustic results within several days. The possibility to use multi-GPU workstation computers rather than supercomputing facilities to speed up time-to-solution and simplify the CFD process is certainly a promising area of research.

## 6. Summary

We have attempted to provide a concise overview of the developments in jet LES during the last decade since the review by Bodony & Lele [4]. Much progress has been achieved in terms of meshing capabilities, numerical methods and modelling approaches, and the community using LES for jet noise has expanded considerably. Several complex, multi-physics, computational frameworks have been developed for LES in different research groups including national laboratories, and commercial CFD tools, such as ANSYS Fluent, Star-CCM+, PowerFlow, etc. are also being applied. We also highlighted areas where LES is now contributing towards a better understanding of jet flow turbulence and how it is used to complement experiments and guide the jet flow and noise modelling efforts. As surmised in [2,3], jet aeroacoustics research at present is arguably in a second golden age and different application-oriented efforts are also employing LES. It must nevertheless be stressed that further advances in LES modelling and hybrid approaches to modelling a more complete aero-engine are required in the future. The demanding technological goals which drive this research require that quantitative assessments and collaborative efforts are pursued, making effective use of all available tools from theory and modelling (including use of data-centric approaches), to computations, experiments, field- and full-scale testing. The integration between computations and experiments has significantly intensified in the last decade and this trend is expected to continue further.

## Data accessibility

This article does not contain any additional data.
